# Impact of Supplemental Nutrition Assistance Program Benefit Reduction or Loss on Food-at-Home Acquisitions and Community Food Program Use

**DOI:** 10.3390/ijerph182212004

**Published:** 2021-11-16

**Authors:** Namrata Sanjeevi

**Affiliations:** Department of Nutritional Sciences, College of Natural Sciences, The University of Texas at Austin, Austin, TX 78712, USA; namratas@utexas.edu

**Keywords:** Supplemental Nutrition Assistance Program, FoodAPS, Healthy Eating Index, community food program, food-at-home acquisitions

## Abstract

Since Supplemental Nutrition Assistance Program (SNAP) benefits are vital for food-at-home (FAH) acquisitions among participating families, changes in participation or benefit amounts may impact FAH purchase and use of community-based food programs (CFP). The association of the loss of or a reduction in SNAP benefits with FAH acquisitions and CFP use was assessed using 2012–2013 National Household Food Acquisition and Purchase Survey data. Households with incomes equal to or below 130% of the Federal Poverty Level were categorized as (1) current SNAP households, (2) households with benefit loss in the preceding year, or (3) households with benefit loss for more than a year. Current SNAP households were classified as receiving (1) lesser-than-usual benefits or (2) usual benefits. Regression analyses examined associations of the loss of or a reduction in benefits with the Healthy Eating Index-2015 (HEI-2015) scores of FAH purchases and CFP use. Benefit loss in the preceding year was related to a lower total HEI-2015 score for FAH acquisitions, whereas benefit reduction was associated with lower green/bean and added sugar scores and increased CFP use. This study suggests that the loss of or a reduction in SNAP benefits may adversely impact the quality of FAH purchases. The findings also suggest that efforts enhancing the nutrition environment of community food sources could support healthy food acquisition by families experiencing benefit reduction.

## 1. Introduction

The Supplemental Nutrition Assistance Program (SNAP), administered by the United States Department of Agriculture (USDA), is the largest nutrition assistance program in the United States; in 2019, SNAP served about 35.7 million Americans and distributed about $130 as the average monthly benefit per person [1]. The benefits distributed by this program reduce poverty and enhance the food purchasing power of participating households [2]. Eligibility guidelines for SNAP participation include a monthly gross income at or below 130% of the Federal Poverty Level (FPL) and a countable asset limit of about $2250 for households with no elderly or disabled members [3]. Further, able-bodied adults without dependents are expected to meet the stipulated work requirements for program qualification [4]. These guidelines collectively suggest that any monthly fluctuations in income, increase in assets, or loss of employment may disqualify income-eligible households from program participation. In place of SNAP disenrollment, some households may experience a reduction in SNAP benefits following a temporary increase in income [5]. Assessing the impact of SNAP disenrollment and benefit reduction on food purchases could be vital for informing strategies that aim to strengthen the program.

Although SNAP disenrollment and benefit reduction may adversely affect household nutrition, only one study has compared the dietary intake of current SNAP participants with that of former participants [6]. Further, no research has examined the impact of SNAP benefit reduction on the dietary intake of household members. Since SNAP benefits are distributed at the household level and are predominantly used for food-at-home (FAH) purchases [7], the use of grocery acquisition data as an alternative source of dietary data could enhance understanding of the impact of SNAP disenrollment and benefit reduction on household nutrition.

Previous studies have shown that the loss of or a reduction in SNAP benefits increases food insecurity among household members [5,6,8]. Community-based food assistance programs (CFP), such as food pantries and meal centers, could supplement the diets of food-insecure individuals by distributing foods at no cost [9]. Although the eligibility criteria for CFP may vary by location and type of program [10], several states allow participation provided that the household income is less than or equal to 185% of the FPL [11]. Although CFP could serve as an alternative source of food acquisition for families experiencing the loss of or a reduction in SNAP benefits, no study has examined whether these households have greater CFP use. Understanding this relationship could inform strategies promoting CFP outreach and use by vulnerable families.

The goal of this study is to examine the relationship of the loss of or a reduction in SNAP benefits with the nutritional quality of FAH purchases and CFP use by leveraging the 2012–2013 National Household Food Acquisition and Purchase Survey (FoodAPS) data.

## 2. Methods

### 2.1. Data Source and Study Population

The current research used the USDA FoodAPS study that provides comprehensive data on food acquisitions and purchases by U.S. households [12]. FoodAPS data were collected during 2012–2013 from a nationally representative sample of 4826 households using a multistage sampling design. The primary respondent, defined as the main food shopper or meal planner, answered questions related to sociodemographic characteristics, SNAP participation, and CFP use during the initial household visit [13]. The primary respondent was also trained to use food books and hand scanners to record food acquisitions over a 1-week period during the initial interview and was responsible for training any household members. Purchasing and acquisitions are classified to indicate FAH and food-away-from-home (FAFH) events. Since SNAP benefits are used for FAH acquisitions [7], the present study focused only on FAH purchases (i.e., groceries, foods, and drinks that are brought home and used to prepare meals for at-home or outside consumption, such as at work). Missing quantities for FAH items were imputed by FoodAPS researchers using information on food items, price, purchase venue, and household characteristics [14]. Detailed information on FoodAPS survey design and data collection are described elsewhere [15]. The FoodAPS data were publicly available and fully anonymized. The institutional review board of the survey contractor, Mathematica Policy Research, approved the FoodAPS protocol.

### 2.2. Analytical Sample

Of the 4826 surveyed FoodAPS households, 4367 households had data on FAH acquisitions. Similar to previous studies [16,17], FAH acquisitions consisting of <6 items or >150 items were considered unlikely to represent weekly food purchases; subsequently, households reporting such acquisitions were excluded (*n* = 399). Households with missing item information (*n* = 6) were also excluded. Additionally, one household with total energy acquired from FAH purchases equal to 2,716,827 kcal was considered as an outlier and excluded from the analyses. Of the remaining 3961 households, those responding affirmatively to the question on anyone in the household reported to be receiving SNAP benefits and having an income less than or equal to 130% of the FPL were classified as current SNAP households (*n* = 803). Households with a negative response on current SNAP participation but answering with a yes to whether anyone in the household had ever received SNAP benefits were defined as former participants (*n* = 613). Based on the response to the question on whether anyone in the household had received SNAP benefits in the preceding 12 months, former participating households with incomes less than or equal to 130% of the FPL (*n* = 202) were divided into two groups: (1) income-eligible households with benefit loss in the past year (*n* = 72) and (2) income-eligible households with benefit loss for more than a year (*n* = 130).

Current SNAP households, regardless of household income, with the last reported benefit amounts less than the usual amounts were defined as experiencing a reduction in benefits (*n* = 96), whereas those reporting the usual amounts were classified as not having a change in benefits (*n* = 973).

For analyses using CFP utilization as the outcome, exclusion criteria related to FAH acquisitions were not applied. The resulting sample sizes were 1034 and 244 for current and former SNAP households with incomes less than or equal to 130% of the FPL, respectively. Sample sizes were 1226 and 129 for SNAP households receiving usual and lower-than-usual benefits, respectively.

### 2.3. Diet Quality of FAH Purchases

The Healthy Eating Index-2015 (HEI-2015) was used to measure diet quality, with higher scores reflecting greater adherence of household FAH purchases to the 2015–2020 Dietary Guidelines for Americans [18]. The HEI-2015 consists of nine adequacy components (i.e., total fruits, including whole fruits and 100% fruit juice; whole fruits; total vegetables; greens and beans; whole grains; dairy; total protein foods; seafood and plant proteins; and fatty acids) and four moderation components (i.e., refined grains, sodium, added sugars, and saturated fat). Moderation components are reverse coded such that lower intakes receive higher scores. Publicly available code from the Division of Cancer Control and Population Sciences of the National Cancer Institute was used to calculate the HEI-2015 scores [19]. The HEI-2015 total score was obtained by summing scores across all 13 components, with a maximum possible score of 100.

### 2.4. Use of CFP

A household was categorized as using community food sources if anyone in the household was receiving USDA foods from a local program or distribution site, was receiving meals at home from community programs, had received meals at a community center in the preceding month, or had gone to a food bank or food pantry in the preceding 30 days for groceries.

### 2.5. Covariates

Sociodemographic characteristics considered were the primary respondent’s age, sex, body mass index, race/ethnicity (i.e., non-Hispanic white, non-Hispanic black, Hispanic, or other), and educational attainment (less than or equal to high school or greater than high school). Consistent with the information available in publicly available data, the approximate midpoint of the individual’s age group was used. Additional covariates included household size, rurality, tobacco use, and number of days since receipt of SNAP benefits.

### 2.6. Statistical Analysis

Statistical analyses were performed using SAS (version 9.4; SAS Institute, Inc., Cary, NC, USA) software. The FoodAPS sample weights were used to adjust for differential selection probabilities and nonresponse. The Taylor series linearization method was used for variance estimation in all analyses. Skewness and kurtosis indices were used to assess normality [20]. Linear regression (for continuous variables) and chi-square tests (for categorical variables) examined demographic differences by SNAP participation status and benefit amount. Linear regression analyses estimated the relationship of loss of SNAP benefits with HEI-2015 total and component scores of FAH purchases, adjusting for the primary respondent’s age, sex, race/ethnicity, body mass index, education level, household size, rurality, WIC participation, and tobacco use. Linear regression analyses estimating the association between reduction of SNAP benefits and HEI-2015 scores were additionally adjusted for the number of days since the receipt of benefits.

Logistic regression analyses examined associations of the loss of or a reduction in SNAP benefits with the odds of CFP use. Analyses using loss of SNAP benefits as an independent variable were adjusted for the primary respondent’s age, sex, race/ethnicity, body mass index, education level, household size, rurality, WIC participation, and tobacco use. Analyses using a reduction in SNAP benefits as an independent variable were additionally adjusted for the number of days since the receipt of benefits. A *p* < 0.05 was used to indicate statistical significance.

## 3. Results

### 3.1. Demographic Characteristics

Table 1 and Table 2 indicate the demographic characteristics of households by SNAP participation status and benefit amount. The age of the primary respondent was significantly lower in former households with benefits cut off in the preceding year but higher in households with benefits cut off for more than a year. Households experiencing benefit loss for more than a year had a significantly lower household size than current SNAP families. A greater proportion of households receiving less-than-usual benefit amounts were from non-rural areas.

### 3.2. Loss of Snap Benefits and Hei-2015 Scores of Fah Purchases

The mean ± the standard error of HEI-2015 total scores for current participants, former participants with benefit loss in the preceding year, and former participants with benefit loss for more than a year were 49.0 ± 0.7, 45.8 ± 1.7, and 48.7 ± 2.1. Former participants with benefits cut off in the preceding year had a significantly lower HEI-2015 total score than current participants (Table 3). Loss of benefits in the preceding year was also associated with lower total and whole-fruit scores. Further, loss of benefits for more than a year was related to significantly lower whole grain scores. Other HEI-2015 component scores did not significantly differ between former and current participants.

### 3.3. Reduction in Snap Benefits and Hei-2015 Scores of Fah Purchases

The mean ± the standard error of HEI-2015 total scores for participants receiving the same benefit amounts and less-than-usual amounts were 49.5 ± 0.6 and 48.8 ± 1.5. A reduction in SNAP benefits was not significantly associated with HEI-2015 total scores of FAH purchases (Table 4). However, households receiving less-than-usual benefit amounts had significantly lower green and bean and added sugar scores but higher refined grain scores than those receiving the usual amounts. A reduction in the benefit amount was also associated with lower whole-fruit scores; however, this association was only marginally significant.

### 3.4. Loss of or a Reduction in SNAP Benefits and CFP Use

Compared to current SNAP households, those with benefits cut off in the preceding year did not have significantly higher odds of CFP use (OR (95% CI) = 0.56 (0.26,1.20); data not shown in table). Benefit loss for more than a year was also not associated with CFP use (OR (95% CI) = 0.73 (0.41,1.30)). However, households receiving less-than-usual benefit amounts had significantly greater odds of CFP use (OR (95% CI) = 2.24 (1.47,3.42)) than those receiving the usual amounts.

## 4. Discussion

The current study found that income-eligible households experiencing loss of benefits in the preceding year had a significantly lower diet quality of FAH purchases than current SNAP households. Recent loss of benefits was also associated with lower component scores for total and whole fruits. Further, income-eligible households with benefits cut off for more than a year had significantly lower whole-grain scores than current SNAP families. Compared to households receiving the usual benefit amounts, those with benefit reduction had lower scores for greens and beans and added sugars but higher scores for refined grains. Although loss of benefits was not associated with CFP use, households receiving less-than-usual benefit amounts had significantly higher odds of CFP use than those receiving the usual amounts. Taken together, the findings imply suboptimal food purchasing patterns and greater reliance on community food sources among households experiencing a loss of or a reduction in SNAP benefits.

In the current study, about 33% of the former SNAP households met income eligibility for participation (i.e., income greater than of equal to 130% of the FPL). Loss of benefits in the past year was significantly associated with lower diet quality of FAH purchases, as measured by HEI-2015. The current finding differs from a previous NHANES study suggesting lack of association of loss of benefits with overall diet quality in adults and children [6]. This discrepancy may be explained by differences in study methodology; while the previous NHANES study used two 24-h dietary recalls to summarize individual-level FAH and FAFH consumption, the current research focused on household-level FAH purchases during a 1-week period. Loss of benefits in the preceding year was also significantly associated with lower total and whole-fruit scores of FAH purchases. Although loss of benefits for more than a year was not related to the diet quality of FAH purchases, households with benefits cut off for more than a year had significantly lower whole-grain scores than current SNAP households. This finding is somewhat comparable to previous research suggesting lower whole-grain intake among children from households experiencing loss of benefits for more than a year [6]. The findings collectively imply that SNAP plays a vital role in enhancing dietary adequacy of participating households and income-eligible households experiencing benefit loss may have lower acquisitions of healthful foods.

In the current study, households receiving lower-than-usual benefit amounts had higher scores for refined grains compared to those receiving the usual amounts. Since refined grains constitute a predominant portion of household grocery expenses among SNAP families [21], it is possible that a reduction in SNAP benefits may be accompanied by lower spending and subsequent acquisition of refined grains. However, a reduction in SNAP benefits was significantly related to lower scores for greens and beans and added sugars. Further, the association of SNAP benefit reduction with lower whole-fruit scores approached statistical significance. Cost is commonly cited as a barrier toward purchase and consumption of fruits and vegetables by low-income families [22]. Further, previous research has shown that households with budgetary constraints are more likely to consume foods higher in added sugar [23]. A reduction in SNAP benefits may further limit resources available for FAH acquisitions among participating families, thereby exacerbating the trend toward reduced fruit and vegetable and greater added sugar purchase. Although the COVID-19 relief package and revised Thrifty Food Plan have modestly increased SNAP benefits [24], the current findings hold implications regarding benefit reductions that may occur following these changes.

Loss of benefits was not related to CFP use in the current study, with about 24% and 19% of current and former SNAP families reporting CFP use, respectively (data not shown). Previous research suggests that SNAP participants exhibit increased CFP use following exhaustion of benefits [25]; thus, it is likely that current SNAP households could rely on community food sources at a rate comparable to that of former households. However, SNAP families receiving less-than-usual benefit amounts had significantly greater odds of CFP use than those receiving the usual amounts. Since SNAP participation confers automatic eligibility for CFP receipt in several states, an alternate explanation for the lack of relationship of loss of benefits with CFP use could be obstacles related to providing proof of eligibility among former SNAP households [26]. Future research examining barriers toward CFP use could inform strategies that promote use of alternative sources of nutrition among income-eligible former SNAP households.

The limitations of this study must be considered while interpreting the findings. The cross-sectional nature of the study design limits causal inferences. Since the study data were collected between April 2012 and January 2013, FAH acquisitions may not reflect any seasonal variations. Despite the use of a nationally representative sample, the sample size of former SNAP households was small. The possibility of voluntary disenrollment among income-eligible former SNAP households cannot be ruled out. Lack of information on food waste and frequency of CFP use could have confounded some of the associations observed in this study. Further, the recall timeframe for the questions assessing CFP use might not have coincided with the timing of SNAP benefit reduction. Since FoodAPS collects information on food purchases only for a period of 1 week, the data may not reflect intrinsic food purchasing variations that might have occurred during the monthly SNAP benefit cycle. Lack of information on the extent of change in benefit amount could have confounded the relationship of reduction in benefits with FAH acquisitions. Additionally, households were classified as receiving the usual or less-than-usual amounts based on responses at the time of the initial interview. Thus, any changes in benefit amounts during the food reporting week could have resulted in misclassification; however, using information on the number of days since receipt of benefits and households reporting change in benefits, this was estimated to have occurred for less than 1% of the households. Despite these limitations, the use of a nationally representative sample of household food purchases increases the generalizability of the study findings to the U.S. population. Despite an increase in nonresponse over the 1-week data collection period [27], food acquisition information obtained via hand scanning item barcodes should have lower recall error compared to dietary data. The distinction between FAH and FAFH purchases in FoodAPS data facilitated studying the impact of changes in SNAP participation and benefit amount on FAH purchases. Future research that examines food purchasing decisions among families experiencing benefit loss/reduction could inform policies that target to improve nutrition outcomes in this population.

## 5. Conclusions

The study findings imply that loss of benefits could have an adverse impact on the overall quality of FAH acquisitions among income-eligible former SNAP households. The results also suggest that a reduction in benefits could be related to suboptimal acquisitions of certain food groups, including greens and beans and added sugars, and increased CFP use. Thus, efforts to protect families from the negative effects of benefit reduction could leverage community food sources as an avenue to improve nutrition in this population.

## Figures and Tables

**Table 1:** Demographic characteristics ^a^ of income-eligible households by participation in the Supplemental Nutrition Assistance Program (SNAP).

Characteristics ^b^	Current SNAP Households	Households with Benefits Cut off in the Preceding Year	Households with Benefits Cut off for >1 Year
	*n* = 1034	*n* = 84	*n* = 160
Primary respondent’s age			
Years	46.8 ± 1.3	39.5 ± 2.1 **	54.3 ± 1.9 **
Primary respondent’s sex			
Male	27.2	25.9	38.4
Female	72.8	74.1	61.6
Primary respondent’s race/ethnicity			
Hispanic	22.9	21.8	22.8
Non-Hispanic white	44.3	51.3	48.3
Non-Hispanic black	27.3	23.7	23.2
Other race	5.6	3.1	5.7
Household size	2.7 ± 0.1	2.9 ± 0.3	2.1 ± 0.2 **
Primary respondent’s education level			
Less than high school/high school	65.3	72.0	58.0
Some college/college graduate or above	34.7	28.1	42.0
Household tobacco use			
Yes	51.2	45.1	40.4
No	48.8	54.9	59.6
Household rurality			
Rural	30.0	37.6	25.8
Non-rural	70.0	62.4	74.2

^a^ Data are represented as the mean ± the standard error of the mean or %; ^b^ primary respondent’s age and household size were significantly different between groups (using linear regression with current participants as the reference category). ** *p* < 0.01.

**Table 2:** Household demographics ^a^ by Supplemental Nutrition Assistance Program (SNAP) benefit amount.

Characteristics ^b^	Households with Last Benefit Amounts	
	Same as Usual	Less than Usual
	*n* = 1226	*n* = 129
Primary respondent’s age		
Years	46.5 ± 1.1	44.3 ± 1.6
Primary respondent’s sex		
Male	25.6	25.6
Female	74.4	74.4
Primary respondent’s race/ethnicity		
Hispanic	22.5	25.8
Non-Hispanic white	45.4	36.2
Non-Hispanic black	26.6	33.4
Other race	5.5	4.5
Household size	2.8 ± 0.1	3.1 ± 0.2
Primary respondent’s education level		
Less than high school/high school	62.1	56.4
Some college/college graduate or above	37.9	43.6
Household tobacco use		
Yes	52.8	51.9
No	47.2	48.1
Household rurality ***		
Rural	30.6	13.1
Non-rural	69.4	86.9

^a^ Data are represented as the mean ± the standard error of the mean or %; ^b^ household rurality was significantly different between groups (using chi-square test); *** *p* < 0.001.

**Table 3:** Association ^a^ of SNAP ^b^ participation status with HEI ^c^-2015 total and component scores of food-at-home acquisitions among income-eligible households in the National Household Food Acquisition and Purchase Survey 2012–2013 (*n* = 1005).

HEI-2015 Score	Current SNAP Households	Households with Benefits Cut off in the Preceding Year			Households with Benefits Cut off for More than a Year		
		Estimate (SE ^d^)	*p*-Value	95% CI ^e^	Estimate (SE)	*p*-Value	95% CI
Total score	Ref	−3.10 (1.49)	0.046	−6.14, −0.06	−1.35 (2.35)	0.57	−6.14, 3.45
Adequacy components							
Total fruits	Ref	−0.83 (0.21)	0.0004	−1.26, −0.40	−0.13 (0.33)	0.70	−0.79, 0.54
Whole fruits	Ref	−0.87 (0.21)	0.0002	−1.30, −0.45	−0.04 (0.28)	0.89	−0.62, 0.54
Total vegetables	Ref	−0.32 (0.36)	0.38	−1.05, 0.41	−0.20 (0.33)	0.56	−0.87, 0.48
Greens and beans	Ref	0.05 (0.21)	0.82	−0.38, 0.48	−0.24 (0.27)	0.38	−0.80, 0.31
Whole grains	Ref	0.01 (0.43)	0.98	−0.86, 0.88	−0.60 (0.30)	0.0496	−1.20, −0.001
Dairy	Ref	−0.27 (0.86)	0.76	−2.03, 1.49	−0.08 (0.40)	0.85	−0.90, 0.74
Total protein foods	Ref	0.08 (0.27)	0.78	−0.48, 0.63	−0.14 (0.23)	0.54	−0.62, 0.33
Seafood and plant proteins	Ref	−0.36 (0.27)	0.19	−0.90, 0.19	0.19 (0.28)	0.51	−0.38, 0.76
Fatty acids	Ref	0.04 (0.86)	0.96	−1.70, 1.79	0.16 (0.71)	0.82	−1.28, 1.60
Moderation components							
Refined grains	Ref	−0.22 (0.70)	0.75	−1.65, 1.21	−0.57 (0.71)	0.43	−2.02, 0.87
Sodium	Ref	−0.78 (0.80)	0.34	−2.41, 0.85	0.56 (0.48)	0.25	−0.41, 1.54
Saturated fats (% kcal)	Ref	−0.41 (0.61)	0.51	−1.65, 0.83	−0.22 (0.42)	0.60	−1.08, 0.63
Added sugars (% kcal)	Ref	0.78 (0.55)	0.16	−0.33, 1.90	−0.03 (0.74)	0.97	−1.54, 1.48

^a^ Model adjusted for the primary respondent’s age, sex, race/ethnicity, body mass index, and education level, household size, rurality, WIC participation, and tobacco use; ^b^ SNAP, Supplemental Nutrition Assistance Program; ^c^ HEI, Healthy Eating Index; ^d^ standard error; ^e^ confidence intervals.

**Table 4:** Association ^a^ of benefit reduction with HEI ^b^-2015 total and component scores of food-at-home purchases among SNAP ^c^ households in the National Household Food Acquisition and Purchase Survey 2012–2013 (*n* = 1069).

HEI-2015 Score	Households with Last Benefit Amount			
	Same as Usual	Less than Usual		
		Estimate (SE ^d^)	*p*-Value	95% CI ^e^
Total score	Ref	−1.05 (1.62)	0.52	−4.35, 2.25
Adequacy components				
Total fruits	Ref	−0.19 (0.24)	0.45	−0.68, 0.31
Whole fruits	Ref	−0.45 (0.26)	0.09	−0.98, 0.07
Total vegetables	Ref	−0.15 (0.29)	0.60	−0.75, 0.44
Greens and beans	Ref	−0.64 (0.19)	0.002	−1.02, −0.27
Whole grains	Ref	0.22 (0.47)	0.63	−0.73, 1.18
Dairy	Ref	−1.00 (0.62)	0.12	−2.26, 0.26
Total protein foods	Ref	−0.08 (0.24)	0.74	−0.58, 0.42
Seafood and plant proteins	Ref	0.10 (0.29)	0.73	−0.49, 0.69
Fatty acids	Ref	0.42 (0.55)	0.46	−0.71, 1.55
Moderation components				
Refined grains	Ref	1.36 (0.43)	0.003	0.49, 2.24
Sodium	Ref	0.62 (0.71)	0.39	−0.82, 2.06
Saturated fats (% kcal)	Ref	0.05 (0.63)	0.93	−1.23, 1.34
Added sugars (% kcal)	Ref	−1.31 (0.57)	0.03	−2.47, −0.14

^a^ Model adjusted for the primary respondent’s age, sex, race/ethnicity, body mass index, and education level, household size, WIC participation, rurality, tobacco use, and the number of days since receipt of benefits; ^b^ HEI, Healthy Eating Index; ^c^ SNAP, Supplemental Nutrition Assistance Program; ^d^ standard error.; ^e^ confidence intervals.
